# Supplementary material for: Cdc48-like protein of actinobacteria (Cpa) is a novel proteasome interactor in mycobacteria and related organisms
Source: eLife. 2018 May 29;7:e34055. doi: 10.7554/eLife.34055 (PMC6017811; doi:10.7554/eLife.34055)
Supplement: Supplementary file 1. — Supplementary Table 1. List of proteins used for ClustalO alignment of the Cdc48 family (NCBI accession numbers). Supplementary Table 2. List of proteins accumulating during growth in the presence of glycerol in M. smegmatis Δcpa as compared to its parent strain by label-free quantification mass spectrometry. Supplementary Table 3. List of proteins depleted during growth in the presence of glycerol in M. smegmatis Δcpa as compared to its parent strain by label-free quantification mass spectrometry. Supplementary Table 4. List of proteins accumulating during growth in the absence of glycerol in M. smegmatis Δcpa as compared to its parent strain by label-free quantification mass spectrometry. Supplementary Table 5. List of proteins depleted during growth in the absence of glycerol in M. smegmatis Δcpa as compared to its parent strain by label-free quantification mass spectrometry. [file elife-34055-supp1.docx]

**Supplementary Table 1**

List of proteins used for ClustalO alignment of the Cdc48 family (NCBI accession numbers).

| WP_018682511 |
| --- |
| WP_018332384 |
| WP_020513115 |
| WP_014447816 |
| WP_017973061 |
| WP_012783062 |
| WP_020631894 |
| WP_005160576 |
| WP_020641740 |
| WP_020664411 |
| WP_013805367 |
| WP_010540887 |
| WP_017837830 |
| WP_005171895 |
| WP_006358093 |
| WP_006436878 |
| WP_012832586 |
| WP_008379603 |
| WP_007240178 |
| WP_007623749 |
| WP_018351876 |
| WP_013288870 |
| WP_007465870 |
| WP_007073278 |
| WP_005092266 |
| WP_013988744 |
| WP_011726205 |
| WP_019283953 |
| WP_014000230 |
| WP_014813835 |
| WP_007768380 |
| WP_011891157 |
| WP_005624475 |
| WP_009952556 |
| WP_015354415 |
| WP_012392700 |
| WP_007167568 |
| WP_003887070 |
| WP_005143261 |
| WP_015304514 |
| WP_003901853 |
| WP_011739550 |
| WP_003932831 |
| WP_011778023 |
| WP_003923299 |
| WP_019049628 |
| WP_014988722 |
| WP_014353308 |
| WP_011211823 |
| WP_013677737 |
| WP_019744483 |
| WP_007301324 |
| WP_012689081 |
| WP_006553634 |
| WP_003941536 |
| WP_010836706 |
| WP_003937423 |
| WP_007534951 |
| WP_005441493 |
| WP_005459614 |
| WP_005466490 |
| WP_009156361 |
| WP_015787726 |
| WP_006239014 |
| WP_009950923 |
| WP_010308459 |
| WP_015097912 |
| WP_018800055 |
| WP_018812401 |
| WP_011904175 |
| WP_020502169 |
| WP_013139040 |
| WP_018158680 |
| WP_013015722 |
| WP_013128285 |
| WP_013736188 |
| WP_008086210 |
| WP_008086490 |
| WP_015283696 |
| WP_018204475 |
| WP_009886954 |
| WP_007983710 |
| WP_008414326 |
| WP_020222052 |
| WP_008311543 |
| WP_005538167 |
| WP_014040841 |
| WP_004591599 |
| WP_011223677 |
| WP_004518130 |
| WP_009760720 |
| WP_010903216 |
| WP_007141998 |
| WP_010546782 |
| WP_006671061 |
| WP_007692403 |
| WP_006079005 |
| WP_005045463 |
| WP_007741520 |
| WP_004969704 |
| WP_008325984 |
| WP_007542766 |
| WP_004059798 |
| WP_008318315 |
| WP_004975353 |
| WP_004042269 |
| WP_008605595 |
| WP_007274530 |
| WP_006053635 |
| WP_009376173 |
| WP_018259072 |
| WP_015762736 |
| WP_014051352 |
| WP_013878602 |
| WP_011572382 |
| WP_008526670 |
| WP_015790342 |
| WP_007999876 |
| WP_008443672 |
| WP_006112021 |
| WP_004598482 |
| WP_008585645 |
| WP_008848399 |
| WP_015910437 |
| WP_008008290 |
| WP_004046396 |
| WP_017344655 |
| WP_006627744 |
| WP_008384642 |
| WP_006884882 |
| WP_008013962 |
| WP_006649349 |
| WP_012943348 |
| WP_007704005 |
| WP_015301226 |
| WP_004030208 |
| WP_013645634 |
| WP_013825116 |
| WP_012955237 |
| WP_011954019 |
| WP_004032397 |
| WP_004036511 |
| WP_019262409 |
| WP_019264538 |
| WP_019266466 |
| WP_016357855 |
| WP_015791307 |
| WP_013099461 |
| WP_010870669 |
| WP_012980381 |
| WP_004590655 |
| WP_015733284 |
| WP_012036428 |
| WP_014404837 |
| WP_012901561 |
| WP_011499714 |
| WP_011500052 |
| WP_011833000 |
| WP_014867260 |
| WP_011844989 |
| WP_004037537 |
| WP_013194481 |
| WP_013195568 |
| WP_013036726 |
| WP_007314790 |
| WP_015053218 |
| WP_015053913 |
| WP_019178588 |
| WP_015323967 |
| WP_015325606 |
| WP_004077524 |
| WP_013329826 |
| WP_012108004 |
| WP_015284270 |
| WP_013719008 |
| WP_013720278 |
| WP_014586984 |
| WP_014587553 |
| WP_011695278 |
| WP_011695801 |
| WP_013897698 |
| WP_013899198 |
| WP_011023443 |
| WP_011024448 |
| WP_011305931 |
| WP_011307291 |
| WP_011032400 |
| WP_011033205 |
| WP_011406666 |
| WP_012616965 |
| WP_011449410 |
| WP_013295059 |
| WP_010877247 |
| WP_013414045 |
| WP_006666688 |
| WP_006108995 |
| WP_006168140 |
| WP_006651486 |
| WP_004213591 |
| WP_006826008 |
| WP_006184308 |
| WP_006179730 |
| WP_008453525 |
| WP_006431295 |
| WP_005580623 |
| WP_005559314 |
| WP_008419917 |
| WP_015322678 |
| WP_007259092 |
| WP_011323249 |
| WP_006067722 |
| WP_008164746 |
| WP_006088250 |
| WP_011177257 |
| WP_010901251 |
| WP_010917205 |
| WP_008441988 |
| WP_046868385.1 |
| ANY77037.1 |
| WP_047188466.1 |
| WP_037503572.1 |
| WP_029941576.1 |
| WP_037530033.1 |
| WP_010409860.1 |
| KPM24385.1 |
| KTS12597.1 |
| WP_043832551.1 |
| WP_075797656.1 |
| WP_047004176.1 |
| WP_034906555.1 |
| WP_048884782.1 |
| WP_051489262.1 |
| WP_051491873.1 |
| WP_034510147.1 |
| WP_028747596.1 |
| WP_041935807.1 |
| WP_010109842.1 |
| WP_066571810.1 |
| WP_011769640.1 |
| WP_011552840.1 |
| WP_090488903.1 |
| WP_011939689.1 |
| WP_006562814.1 |
| WP_007906072.1 |
| WP_004569100.1 |
| WP_013275281.1 |
| APC08799.1 |
| CEP67328.1 |
| WP_062283753.1 |
| WP_085544635.1 |
| WP_005659447.1 |
| WP_013047776.1 |
| WP_078016087.1 |
| ABH06968.1 |
| JAV44339.1 |
| KIH62580.1 |
| EFH46991.1 |
| EFH51078.1 |
| ACI33015.1 |
| AGO67238.1 |
| KJH53218.1 |
| XP_005221029.1 |
| EDP07906.1 |
| KTF85885.1 |
| KTF88408.1 |
| KTG34381.1 |
| BAA13101.1 |
| AAO65962.1 |
| AAW58140.1 |
| BAM82129.1 |
| OWR48697.1 |
| AAC48226.1 |
| EAL51407.1 |
| XP_656792.1 |
| AAA83413.1 |
| AAC46844.1 |
| AAA75044.1 |
| AAF54995.2 |
| P54351.2 |
| AHN57317.1 |
| AHN57318.1 |
| EZG52949.1 |
| AAH06627.1 |
| BAC33656.1 |
| P46460.2 |
| EDL34203.1 |
| EDL34204.1 |
| NP_006169.2 |
| BAF82893.1 |
| EEB10550.1 |
| EEB10551.1 |
| XP_002423289.1 |
| AAU44261.1 |
| BAF17977.1 |
| BAG93228.1 |
| NP_001153385.1 |
| CCA39825.1 |
| EDL43325.1 |
| XP_004868726.1 |
| NP_001297212.1 |
| AAD39485.1 |
| EDM06287.1 |
| EDM06288.1 |
| KHJ96356.1 |
| CAC87940.1 |
| KNH03807.1 |
| NP_001231489.1 |
| XP_005668753.1 |
| XP_013836318.1 |
| KHJ44108.1 |
| GAB66301.1 |
| XP_001351105.1 |
| KMZ86838.1 |
| KMZ80761.1 |
| KMZ93268.1 |
| KMZ99832.1 |
| XP_001613052.1 |
| AAG17479.1 |
| XP_011663388.1 |
| DAA07199.1 |
| NP_009636.3 |
| ANM86150.1 |
| ANB15066.1 |
| CAH93506.1 |
| AAD17345.1 |
| CAB81033.1 |
| NP_192400.2 |
| EAN32453.1 |
| XP_764736.1 |
| AAF18300.1 |
| EDV26431.1 |
| NP_001072788.1 |
| XP_012808037.1 |
| CAJ16140.1 |
| XP_001218858.1 |
| CBH08889.1 |
| KEG11712.1 |
| AAQ83118.1 |
| AAY44601.1 |
| XP_005156264.1 |
| XP_017213950.1 |
| XP_021325418.1 |
| BAA85162.1 |
| NP_082053.1 |
| NP_001280735.1 |
| Q5BL07.2 |
| AAB87880.1 |
| JAV39524.1 |
| AAB99758.1 |
| ABS71030.1 |
| XP_022283051.1 |
| XP_022283050.1 |
| XP_013974518.1 |
| XP_013974516.1 |
| XP_013974515.1 |
| XP_013974514.1 |
| XP_532459.1 |
| XP_022054393.1 |
| NP_001164306.1 |
| XP_021791740.1 |
| XP_017812457.1 |
| XP_009201693.1 |
| XP_009201689.1 |
| XP_009201688.1 |
| XP_009201687.1 |
| XP_003896383.1 |
| XP_021576065.1 |
| XP_021576064.1 |
| XP_005340598.1 |
| XP_021562572.1 |
| XP_021529890.1 |
| XP_021529889.1 |
| XP_021529888.1 |
| XP_021529887.1 |
| XP_021529886.1 |
| XP_021545202.1 |
| XP_021417786.1 |
| XP_021407342.1 |
| XP_021407340.1 |
| XP_021407339.1 |
| OWK59680.1 |
| XP_021244832.1 |
| XP_021244830.1 |
| XP_021171691.1 |
| XP_012717000.1 |
| XP_021135275.1 |
| XP_021135272.1 |
| XP_021098244.1 |
| XP_004862425.1 |
| XP_021090632.1 |
| XP_021017515.1 |
| XP_021017514.1 |
| XP_021017513.1 |
| XP_021017512.1 |
| XP_021045904.1 |
| XP_021045902.1 |
| XP_020958039.1 |
| XP_003357485.3 |
| XP_020788252.1 |
| XP_020640891.1 |
| XP_020640890.1 |
| XP_020562955.1 |
| XP_011479493.1 |
| XP_011479492.1 |
| XP_011479491.1 |
| XP_011479490.1 |
| XP_020462508.1 |
| XP_020487237.1 |
| XP_010397469.2 |
| OPJ81819.1 |
| XP_020355649.1 |
| XP_012635085.1 |
| XP_012635084.1 |
| XP_020016773.1 |
| XP_020016772.1 |
| NP_001085441.1 |
| SBP51830.1 |
| SBQ97335.1 |
| SBP03344.1 |
| SBP71482.1 |
| SBS58096.1 |
| SBR94703.1 |
| EDL14609.1 |
| KYO26656.1 |
| KYO26655.1 |
| EAW76844.1 |
| EAW76842.1 |
| EAW76841.1 |
| EAW76840.1 |
| EAL24149.1 |
| AFE78731.1 |
| AAH90845.1 |
| AAH35575.1 |
| JAB43810.1 |
| DAA30802.1 |
| JAA35240.1 |
| NP_001179000.1 |
| Q13608.2 |
| OXB84728.1 |
| OXB53718.1 |
| Q99LC9.1 |
| OBS66348.1 |
| JAR61352.1 |
| JAQ35883.1 |
| JAQ31315.1 |
| OWK13242.1 |
| BAA33544.1 |
| Q13608.2 |
| Q99LC9.1 |
| NP_663463.1 |
| AAF62564.1 |
| BAB83047.1 |
| XP_005627423.1 |
| XP_538926.2 |
| XP_022065603.1 |
| XP_022065602.1 |
| XP_021792987.1 |
| XP_021792986.1 |
| XP_021792985.1 |
| XP_009203462.1 |
| XP_009203461.1 |
| XP_021577571.1 |
| XP_021577570.1 |
| XP_008064331.1 |
| XP_008064330.1 |
| XP_021547639.1 |
| XP_012304099.1 |
| XP_021495871.1 |
| XP_021423924.1 |
| XP_021390560.1 |
| OWK54199.1 |
| XP_001332652.5 |
| XP_021245134.1 |
| XP_012708934.1 |
| XP_021115456.1 |
| XP_021115455.1 |
| XP_005072420.1 |
| XP_021005235.1 |
| XP_021073399.1 |
| XP_021073398.1 |
| XP_013833373.1 |
| P54777.1 |
| XP_020832509.1 |
| XP_020787112.1 |
| XP_020787111.1 |
| XP_020787110.1 |
| XP_020769718.1 |
| XP_020664010.1 |
| XP_011472050.1 |
| OPJ81688.1 |
| OPJ81687.1 |
| NP_001027403.2 |
| NP_001163114.1 |
| XP_020337786.1 |
| XP_012628615.1 |
| XP_012628613.1 |
| XP_020012845.1 |
| XP_020012844.1 |
| SBP42316.1 |
| SBR71178.1 |
| SBQ49352.1 |
| SBP16328.1 |
| SBP24808.1 |
| CDG70158.1 |
| EDL23533.1 |
| KYO22267.1 |
| KXJ13397.1 |
| EAX04129.1 |
| EAX04127.1 |
| AFJ70323.1 |
| AAH48331.1 |
| AAH03424.1 |
| AHH37258.1 |
| DAA16590.1 |
| JAA40890.1 |
| CAN13170.1 |
| CAN13169.1 |
| BAD51975.1 |
| BAB83046.1 |
| EZA51687.1 |
| AAM00262.1 |
| EDS43334.1 |
| ETN62319.1 |
| JAP82527.1 |
| JAN87172.1 |
| JAN10037.1 |
| JAM91697.1 |
| JAL78434.1 |
| JAL63174.1 |
| JAK76766.1 |
| JAK58428.1 |
| JAI89931.1 |
| EZA51421.1 |
| AAM43608.1 |
| NP_001332785.1 |
| NP_660208.2 |
| NP_001297402.1 |
| NP_001156983.1 |
| NP_067318.2 |
| NP_001273721.1 |
| NP_001159743.1 |
| NP_001028444.1 |
| XP_008058217.1 |
| XP_020921131.1 |
| XP_021533908.1 |
| XP_021505808.1 |
| XP_021386315.1 |
| XP_021386312.1 |
| XP_021386311.1 |
| XP_021085898.1 |
| XP_021038240.1 |
| XP_021386317.1 |
| XP_021386316.1 |
| OWK55512.1 |
| XP_021234950.1 |
| XP_021234940.1 |
| XP_021234932.1 |
| XP_021141903.1 |
| XP_021141901.1 |
| XP_021141900.1 |
| XP_021141899.1 |
| XP_005501496.2 |
| XP_021130160.1 |
| XP_021130159.1 |
| XP_021130158.1 |
| XP_021130157.1 |
| XP_021092007.1 |
| XP_021092006.1 |
| XP_021092005.1 |
| XP_021038243.1 |
| XP_021059267.1 |
| XP_021059265.1 |
| XP_020852827.1 |
| XP_020852824.1 |
| XP_020852823.1 |
| XP_020750291.1 |
| XP_020750290.1 |
| XP_020666729.1 |
| XP_020666728.1 |
| XP_020666723.1 |
| XP_020666722.1 |
| XP_020666721.1 |
| XP_020666718.1 |
| OPJ81131.1 |
| OPJ81130.1 |
| XP_013962888.1 |
| XP_022198365.1 |
| XP_009189907.1 |
| XP_009189906.1 |
| XP_005337635.2 |
| XP_012296828.1 |
| XP_012296824.1 |
| XP_021329454.1 |
| XP_021329453.1 |
| XP_013222129.1 |
| XP_012957909.1 |
| XP_005022629.1 |
| XP_004854892.1 |
| XP_020852826.1 |
| XP_020852825.1 |
| XP_020449725.1 |
| XP_020449724.1 |
| XP_020449723.1 |
| XP_010398794.1 |
| XP_010398793.1 |
| XP_010398792.1 |
| XP_020285856.1 |
| XP_020285855.1 |
| XP_020285854.1 |
| XP_012592292.1 |
| XP_012592288.1 |
| XP_012592287.1 |
| JAQ36305.1 |
| KYO40424.1 |
| JAQ18534.1 |
| EPQ07571.1 |
| EMP36409.1 |
| ELW47592.1 |
| EHH58473.1 |
| AFE77153.1 |
| KFO22198.1 |
| XP_015909481.1 |
| KZC05261.1 |
| KYQ46404.1 |
| JAR44300.1 |
| KYN33985.1 |
| KYN18422.1 |
| KYN03846.1 |
| KYM82004.1 |
| KOX69925.1 |
| KOC64037.1 |
| KKF16928.1 |
| EKC41223.1 |
| EFN67622.1 |
| Q8NB90.3 |
| Q3UMC0.2 |
| NP_001078921.2 |
| NP_056482.2 |
| P0C874.1 |
| NP_997299.2 |
| Q6ZUB0.1 |
| NP_084323.2 |
| NP_001008360.2 |
| XP_021249686.1 |
| XP_013972877.2 |
| XP_021783278.1 |
| OXA42654.1 |
| NP_001001670.1 |
| XP_021396630.1 |
| NP_001159609.1 |
| XP_021125194.1 |
| NP_849150.3 |
| NP_001138596.1 |
| NP_001138669.1 |
| NP_001337907.1 |
| NP_001102019.1 |
| ELW48713.1 |
| EKC24467.1 |
| NP_001076593.1 |
| NP_001107013.1 |
| XP_012639629.1 |
| KXJ16680.1 |
| EPQ07294.1 |
| EKC42751.1 |
| XP_020557438.1 |
| XP_012639630.1 |
| XP_012639627.1 |
| XP_020027852.1 |
| XP_020027844.1 |
| JAV44521.1 |
| KKF30126.1 |
| EMP38581.1 |
| NP_082433.2 |
| NP_808379.2 |
| XP_013976902.1 |
| XP_005631887.1 |
| XP_540960.2 |
| XP_021794469.1 |
| XP_021794468.1 |
| XP_021794467.1 |
| XP_021794466.1 |
| XP_021794465.1 |
| NP_001138668.1 |
| XP_013218897.2 |
| XP_021571172.1 |
| XP_021537150.1 |
| XP_021537149.1 |
| XP_012328428.1 |
| XP_012328427.1 |
| XP_021503451.1 |
| XP_021487981.1 |
| XP_021487979.1 |
| XP_021439722.1 |
| XP_021410642.1 |
| XP_021410641.1 |
| XP_021410640.1 |
| OWK58622.1 |
| XP_012734671.1 |
| XP_021154338.1 |
| XP_021154337.1 |
| XP_021154336.1 |
| XP_005510742.2 |
| XP_021154335.1 |
| XP_021154334.1 |
| XP_021092122.1 |
| XP_012931346.1 |
| XP_012931344.1 |
| XP_004855003.1 |
| XP_004855002.1 |
| XP_021082562.1 |
| XP_021082561.1 |
| XP_005069750.1 |
| XP_005069749.1 |
| XP_005069748.1 |
| XP_021013422.1 |
| XP_021051544.1 |
| XP_021051543.1 |
| XP_021051542.1 |
| XP_021071795.1 |
| XP_020745157.1 |
| XP_020745152.1 |
| XP_020745147.1 |
| XP_020745138.1 |
| XP_020745130.1 |
| XP_020637203.1 |
| XP_020440548.1 |
| XP_020358105.1 |
| JAV38618.1 |
| JAR74838.1 |
| JAR34495.1 |
| KYO47390.1 |
| KQK77766.1 |
| ELK30919.1 |
| ELK07638.1 |
| EHB17409.1 |
| EGW04169.1 |
| KFO36789.1 |
| XP_022265745.1 |
| XP_022265744.1 |
| XP_022265743.1 |
| XP_022261670.1 |
| XP_022278810.1 |
| XP_022269923.1 |
| XP_022276511.1 |
| XP_022276507.1 |
| XP_022276491.1 |
| XP_013972882.1 |
| XP_005618014.1 |
| XP_005618013.1 |
| XP_005616881.1 |
| XP_005616004.1 |
| XP_005615835.1 |
| XP_022225053.1 |
| XP_022223993.1 |
| XP_022180678.1 |
| XP_022180677.1 |
| XP_022119282.1 |
| XP_022045414.1 |
| XP_021917223.1 |
| XP_021917212.1 |
| XP_021917204.1 |
| XP_021794470.1 |
| XP_003899212.3 |
| XP_001652994.2 |
| OXA60666.1 |
| XP_021527778.1 |
| XP_021533614.1 |
| XP_021533613.1 |
| XP_021484056.1 |
| XP_021503450.1 |
| XP_004929406.1 |
| XP_021189953.1 |
| XP_021154340.1 |
| XP_012929501.2 |
| XP_021036774.1 |
| XP_021036747.1 |
| XP_021036583.1 |
| XP_021036061.1 |
| XP_021071791.1 |
| XP_021071767.1 |
| XP_021071041.1 |
| XP_021070849.1 |
| XP_005660237.2 |
| XP_020863916.1 |
| XP_020863915.1 |
| XP_020863914.1 |
| XP_020863913.1 |
| XP_020863912.1 |
| XP_020863910.1 |
| XP_020863909.1 |
| XP_020853293.1 |
| XP_020828547.1 |
| XP_020828533.1 |
| XP_020828526.1 |
| XP_020807953.1 |
| XP_020773257.1 |
| XP_020725753.1 |
| XP_020725752.1 |
| XP_012174107.1 |
| XP_012174106.1 |
| XP_012253446.1 |
| XP_004521824.1 |
| XP_019141968.1 |
| XP_019141967.1 |
| XP_020299826.1 |
| XP_020137132.1 |
| XP_012611880.2 |
| XP_012611877.2 |
| XP_012611876.2 |
| XP_012611878.2 |
| XP_020136442.1 |
| XP_012611875.1 |
| XP_012611291.1 |
| XP_012634791.1 |
| XP_012620980.1 |
| XP_012620979.1 |
| CCP72602.1 |
| ODM93871.1 |
| EGI68629.1 |
| EFN78441.1 |
| EFN73096.1 |
| OAD62411.1 |
| OAD59486.1 |
| OAD58474.1 |
| KZC09114.1 |
| KYQ49249.1 |
| JAR67720.1 |
| JAR44301.1 |
| JAR44299.1 |
| JAR13905.1 |
| JAR13904.1 |
| JAR13903.1 |
| JAR13902.1 |
| JAQ34671.1 |
| KYO42110.1 |
| KYO40086.1 |
| KYN40579.1 |
| KYN10935.1 |
| KYM99999.1 |
| KYM82902.1 |
| JAQ16164.1 |
| JAQ00168.1 |
| KRZ94217.1 |
| JAO01559.1 |
| KPJ06844.1 |
| KPJ05514.1 |
| KOX75342.1 |
| KOC69069.1 |
| KOB76442.1 |
| JAI48731.1 |
| JAI28108.1 |
| JAI27174.1 |
| KMQ86150.1 |
| JAI11299.1 |
| ELW62642.1 |
| ELK10475.1 |
| EEB14564.1 |
| JAG84415.1 |
| JAG84414.1 |
| JAG84413.1 |
| JAG84251.1 |
| JAD03645.1 |
| JAC97467.1 |
| JAG30299.1 |
| KDR24192.1 |
| AHH40125.1 |
| JAB51296.1 |
| JAB19327.1 |
| XP_013296353.1 |
| XP_022317981.1 |
| NP_001095217.1 |
| NP_958889.1 |
| XP_021876420.1 |
| XP_021885286.1 |
| XP_019035387.1 |
| XP_016609302.1 |
| XP_016591541.1 |
| XP_018994266.1 |
| XP_022087240.1 |
| XP_022053469.1 |
| NP_033529.3 |
| XP_005326290.1 |
| NP_999445.1 |
| XP_008052863.2 |
| XP_021490080.1 |
| XP_021410369.1 |
| XP_005536694.1 |
| XP_021338827.1 |
| XP_021235202.1 |
| XP_012718967.1 |
| XP_021150674.1 |
| XP_021128050.1 |
| XP_004873975.1 |
| XP_005078870.1 |
| XP_021016058.1 |
| XP_021077868.1 |
| XP_020896400.1 |
| XP_020776543.1 |
| XP_020736074.1 |
| NP_001029466.1 |
| NP_001005677.1 |
| XP_020624905.1 |
| XP_004072466.1 |
| XP_020450077.1 |
| XP_020505543.1 |
| XP_010393380.1 |
| XP_020345743.1 |
| XP_012625527.1 |
| XP_019956568.1 |
| XP_011424321.1 |
| XP_010862756.1 |
| XP_019821682.1 |
| XP_019804498.1 |
| XP_019722481.1 |
| XP_019633404.1 |
| XP_002929233.1 |
| XP_019518880.1 |
| XP_019387431.1 |
| XP_019373583.1 |
| XP_014460872.1 |
| XP_019329918.1 |
| NP_001290278.1 |
| NP_001038129.1 |
| XP_019046918.1 |
| XP_019008256.1 |
| XP_018999322.1 |
| XP_002121499.1 |
| XP_018613886.1 |
| XP_018524097.1 |
| XP_003737268.1 |
| XP_018424557.1 |
| XP_018265233.1 |
| NP_001267410.1 |
| XP_017907601.1 |
| XP_017932736.1 |
| XP_017829869.1 |
| XP_017674551.1 |
| XP_017602121.1 |
| XP_008630025.1 |
| XP_017527460.1 |
| XP_017307754.1 |
| XP_017269273.1 |
| XP_002708056.1 |
| XP_008417014.1 |
| XP_008334946.1 |
| XP_008102421.1 |
| XP_003229140.1 |
| XP_016368413.1 |
| XP_016332713.1 |
| XP_007559307.1 |
| XP_016275569.1 |
| XP_001368198.1 |
| XP_005060493.1 |
| XP_007663018.1 |
| XP_016078481.1 |
| XP_016043492.1 |
| XP_015860057.1 |
| XP_015828041.1 |
| XP_015782527.1 |
| XP_007442861.1 |
| XP_015703838.1 |
| XP_015684035.1 |
| XP_007238029.1 |
| XP_006916090.1 |
| XP_006758604.1 |
| XP_015390504.1 |
| XP_015347881.1 |
| XP_015262300.1 |
| XP_015228567.1 |
| XP_006626794.1 |
| XP_015094424.1 |
| XP_014847743.1 |
| XP_014883150.1 |
| XP_014807998.1 |
| XP_014741527.1 |
| XP_014721589.1 |
| XP_014671341.1 |
| XP_014652931.1 |
| XP_005605631.1 |
| XP_014527107.1 |
| XP_006143272.1 |
| XP_005884071.1 |
| XP_014433876.1 |
| XP_014417389.1 |
| XP_006026624.1 |
| XP_014348516.1 |
| XP_005900818.1 |
| XP_005804058.1 |
| XP_004544553.1 |
| XP_005940186.1 |
| XP_014166883.1 |
| XP_014153442.1 |
| XP_014133551.1 |
| XP_014026218.1 |
| XP_005491787.1 |
| XP_013914467.1 |
| XP_013905124.1 |
| XP_005722313.1 |
| XP_005401088.2 |
| XP_013193531.1 |
| XP_013162884.1 |
| XP_013152555.1 |
| XP_013137365.1 |
| XP_013055945.1 |
| XP_003470895.1 |
| XP_009689731.1 |
| XP_012882081.1 |
| XP_004712450.2 |
| XP_012795306.1 |
| XP_004658407.1 |
| XP_004600271.1 |
| XP_004581076.1 |
| XP_012684025.1 |
| XP_003800301.1 |
| XP_004677812.1 |
| XP_012511800.1 |
| XP_004372361.1 |
| XP_012398475.1 |
| XP_004271441.1 |
| XP_012364387.1 |
| XP_004631941.1 |
| XP_004484027.1 |
| XP_005854857.1 |
| XP_012052250.1 |
| XP_011805872.1 |
| XP_011835482.1 |
| XP_011595702.1 |
| XP_011379755.1 |
| XP_010951108.1 |
| XP_010844417.1 |
| XP_010781840.1 |
| XP_003407348.1 |
| XP_009328695.1 |
| XP_010562520.1 |
| XP_003943864.1 |
| XP_010384123.1 |
| XP_010311548.1 |
| XP_010214692.1 |
| XP_010191101.1 |
| XP_010080461.1 |
| XP_010141346.1 |
| XP_009998000.1 |
| XP_009993942.1 |
| XP_009990193.1 |
| XP_010010465.1 |
| XP_009940998.1 |
| XP_009924180.1 |
| XP_009907223.1 |
| XP_009889870.1 |
| XP_009888047.1 |
| XP_009876491.1 |
| XP_009838057.1 |
| XP_009704177.1 |
| XP_009811315.1 |
| XP_009645568.1 |
| XP_009683678.1 |
| XP_009576597.1 |
| XP_009556134.1 |
| XP_009464637.1 |
| XP_008942536.1 |
| XP_008895778.1 |
| XP_008867855.1 |
| XP_008693442.1 |
| XP_008580633.1 |
| XP_008506742.1 |
| XP_008496433.1 |
| XP_008491450.1 |
| XP_008280227.1 |
| XP_008140165.1 |
| XP_005311744.1 |
| XP_007945216.1 |
| XP_007888898.1 |
| XP_007456006.1 |
| XP_007197160.1 |
| XP_006064386.1 |
| XP_007055980.1 |
| XP_007111656.1 |
| XP_006863128.1 |
| XP_006898592.1 |
| XP_005970087.1 |
| XP_001736564.1 |
| XP_001735005.1 |
| XP_818678.1 |
| XP_009310134.1 |
| NP_001037003.1 |
| NP_495705.1 |
| NP_496273.1 |
| XP_018994267.1 |
| XP_022182279.1 |
| XP_022116798.1 |
| XP_021956669.1 |
| XP_021938568.1 |
| XP_001654680.1 |
| XP_001686709.1 |
| XP_021183018.1 |
| XP_012169580.1 |
| XP_012263050.1 |
| XP_953837.1 |
| XP_020306878.1 |
| XP_020281391.1 |
| XP_019533679.1 |
| XP_005182894.1 |
| XP_011261476.1 |
| XP_019865626.1 |
| XP_019764585.1 |
| XP_011135082.1 |
| XP_018902277.1 |
| XP_018778442.1 |
| XP_018566837.1 |
| XP_018399668.1 |
| XP_018370520.1 |
| XP_018350894.1 |
| XP_018324344.1 |
| XP_018306850.1 |
| XP_018046933.1 |
| XP_017759476.1 |
| XP_017483365.1 |
| XP_017146826.1 |
| XP_016911526.1 |
| XP_006563745.1 |
| XP_001605497.2 |
| XP_001949588.1 |
| XP_015985904.1 |
| XP_966692.1 |
| XP_015658481.1 |
| XP_015595352.1 |
| XP_015512792.1 |
| XP_015428732.1 |
| XP_015365471.1 |
| XP_015175542.1 |
| XP_015111852.1 |
| XP_014601733.1 |
| XP_014481318.1 |
| XP_014364670.1 |
| XP_014292957.1 |
| XP_014235190.1 |
| XP_014215517.1 |
| XP_013808289.1 |
| XP_005143521.1 |
| XP_001568770.1 |
| XP_001007447.2 |
| XP_012538772.1 |
| XP_004177198.1 |
| XP_012339791.1 |
| XP_012271777.1 |
| XP_012248683.1 |
| XP_012230716.1 |
| XP_012064233.1 |
| XP_011700134.1 |
| XP_011643105.1 |
| XP_011561178.1 |
| XP_011499520.1 |
| XP_011299904.1 |
| XP_011196577.1 |
| XP_011167409.1 |
| XP_011049393.1 |
| XP_010993943.1 |
| XP_010703076.1 |
| XP_009951643.1 |
| XP_009501604.1 |
| XP_005715271.1 |
| XP_005710763.1 |
| XP_003074428.1 |
| XP_004031986.1 |
| XP_002141464.1 |
| XP_003865258.1 |
| XP_007876628.1 |

**Supplementary Table 2**

List of proteins accumulating during growth in the presence of glycerol in *M. smegmatis* Δ*cpa* as compared to its parent strain by label-free quantification mass spectrometry.

| **Locus tag** | **p-value (q.mod)** | **log_2_(∆/WT)** |
| --- | --- | --- |
| MSMEG_3242 | 1.11E-02 | 1.983 |
| MSMEG_3137 | 5.66E-03 | 1.883 |
| MSMEG_5523 | 3.30E-04 | 1.779 |
| MSMEG_4711 | 4.21E-03 | 1.625 |
| MSMEG_3950 | 4.16E-03 | 1.558 |
| MSMEG_3272 | 2.65E-02 | 1.388 |
| MSMEG_2058 | 6.80E-03 | 1.317 |
| MSMEG_4304 | 2.77E-02 | 1.288 |
| MSMEG_3188 | 4.84E-02 | 1.213 |
| MSMEG_3271 | 4.06E-03 | 1.196 |
| MSMEG_3170 | 3.17E-04 | 1.185 |
| MSMEG_1191 | 5.15E-04 | 1.130 |
| MSMEG_2476 | 5.47E-03 | 1.122 |
| MSMEG_6184 | 4.84E-02 | 1.107 |
| MSMEG_5568 | 1.30E-03 | 1.097 |
| MSMEG_5932 | 3.59E-02 | 1.065 |
| MSMEG_3581 | 5.47E-03 | 1.059 |
| MSMEG_3394 | 4.58E-02 | 1.049 |
| MSMEG_3262 | 9.50E-04 | 1.031 |
| MSMEG_5750 | 8.05E-03 | 1.031 |
| MSMEG_3742 | 4.87E-02 | 1.005 |
| MSMEG_5664 | 1.08E-03 | 0.989 |
| MSMEG_4572 | 4.87E-02 | 0.955 |
| MSMEG_4272 | 4.87E-02 | 0.941 |
| MSMEG_2661 | 1.96E-03 | 0.924 |
| MSMEG_4349 | 4.37E-03 | 0.916 |
| MSMEG_4762 | 4.37E-02 | 0.910 |
| MSMEG_1544 | 2.51E-03 | 0.909 |
| MSMEG_5576 | 8.05E-03 | 0.897 |
| MSMEG_1264 | 4.36E-02 | 0.881 |
| MSMEG_2392 | 4.18E-02 | 0.861 |
| MSMEG_5201 | 1.57E-02 | 0.856 |
| MSMEI_0386 | 3.21E-02 | 0.851 |
| MSMEG_6741 | 4.66E-02 | 0.824 |
| MSMEG_1875 | 6.47E-03 | 0.803 |
| MSMEG_4298 | 6.49E-03 | 0.798 |
| MSMEG_6314 | 3.72E-02 | 0.778 |
| MSMEG_4114 | 2.30E-02 | 0.763 |
| MSMEG_4231 | 1.57E-02 | 0.756 |
| MSMEG_1924 | 5.47E-03 | 0.731 |
| MSMEG_0215 | 4.25E-02 | 0.723 |
| MSMEG_6863 | 8.63E-03 | 0.711 |
| MSMEG_0491 | 4.58E-02 | 0.708 |
| MSMEG_2773 | 8.05E-03 | 0.691 |
| MSMEG_5291 | 2.65E-02 | 0.677 |
| MSMEG_1062 | 1.57E-02 | 0.645 |
| MSMEG_5478 | 3.17E-02 | 0.613 |
| MSMEG_0114 | 1.57E-02 | 0.598 |
| MSMEG_1884 | 4.58E-02 | 0.590 |

**Supplementary Table 3**

List of proteins depleted during growth in the presence of glycerol in *M. smegmatis* Δ*cpa* as compared to its parent strain by label-free quantification mass spectrometry.

| **Locus tag** | **p-value (q.mod)** | **log_2_(∆/WT)** |
| --- | --- | --- |
| MSMEG_0334 | 2.80E-06 | -5.553 |
| MSMEG_6645 | 8.04E-05 | -5.161 |
| MSMEG_2262 | 1.08E-03 | -4.728 |
| MSMEG_0572 | 3.14E-04 | -4.651 |
| MSMEG_1979 | 6.39E-03 | -4.383 |
| MSMEG_5870 | 3.21E-02 | -4.349 |
| MSMEG_3472 | 2.80E-06 | -4.227 |
| MSMEG_6290 | 2.22E-03 | -3.781 |
| MSMEG_1812 | 3.73E-06 | -3.253 |
| MSMEG_6201 | 1.12E-05 | -3.225 |
| MSMEG_5220 | 9.27E-05 | -3.128 |
| MSMEG_6700 | 1.10E-05 | -3.050 |
| MSMEG_5136 | 4.16E-03 | -2.900 |
| MSMEG_1494 | 2.80E-06 | -2.667 |
| MSMEG_5704 | 2.77E-02 | -2.553 |
| MSMEG_0117 | 2.22E-03 | -2.499 |
| MSMEG_3132 | 2.50E-03 | -2.473 |
| MSMEG_4210 | 4.86E-04 | -2.459 |
| MSMEG_6936 | 3.09E-02 | -2.459 |
| MSMEG_0158 | 1.08E-03 | -2.214 |
| MSMEG_6321 | 4.01E-04 | -1.999 |
| MSMEG_4207 | 4.74E-02 | -1.882 |
| MSMEG_6082 | 3.97E-02 | -1.801 |
| MSMEG_6073 | 2.65E-05 | -1.742 |
| MSMEG_4212 | 2.41E-02 | -1.688 |
| MSMEG_3430 | 8.04E-05 | -1.681 |
| MSMEG_1701 | 2.15E-03 | -1.667 |
| MSMEG_0203 | 9.27E-03 | -1.666 |
| MSMEG_0219 | 5.66E-03 | -1.600 |
| MSMEG_4671 | 6.39E-03 | -1.525 |
| MSMEG_0535 | 4.41E-02 | -1.447 |
| MSMEG_5004 | 2.54E-02 | -1.247 |
| MSMEG_5817 | 3.41E-02 | -1.246 |
| MSMEG_1010 | 6.11E-03 | -1.221 |
| MSMEG_4238 | 2.22E-03 | -1.148 |
| MSMEG_6292 | 1.88E-02 | -1.148 |
| MSMEG_5274 | 2.98E-03 | -1.147 |
| MSMEG_2936 | 4.38E-03 | -1.110 |
| MSMEG_6291 | 9.27E-03 | -0.924 |
| MSMEG_0314 | 2.19E-02 | -0.900 |
| MSMEG_6422 | 6.47E-03 | -0.831 |
| MSMEG_5209 | 2.46E-02 | -0.812 |
| MSMEG_6416 | 4.58E-02 | -0.809 |
| MSMEG_0987 | 1.25E-02 | -0.757 |
| MSMEG_2007 | 1.98E-02 | -0.700 |

**Supplementary Table 4**

List of proteins accumulating during growth in the absence of glycerol in *M. smegmatis* Δ*cpa* as compared to its parent strain by label-free quantification mass spectrometry

| **Locus tag** | **p-value (q.mod)** | **log_2_(∆/WT)** |
| --- | --- | --- |
| MSMEG_2793 | 2.06E-06 | 6.482 |
| MSMEG_6068 | 2.06E-06 | 6.455 |
| MSMEG_3752 | 8.96E-06 | 4.195 |
| MSMEG_4350 | 3.40E-07 | 3.610 |
| MSMEG_3581 | 1.46E-05 | 3.380 |
| MSMEG_6070 | 4.54E-02 | 3.183 |
| MSMEG_1325 | 1.55E-02 | 2.954 |
| MSMEG_0911 | 1.16E-05 | 2.643 |
| MSMEG_3249 | 6.05E-03 | 2.420 |
| MSMEG_5487 | 2.30E-03 | 2.336 |
| MSMEG_2691 | 1.71E-06 | 2.318 |
| MSMEG_6190 | 6.78E-03 | 2.315 |
| MSMEG_1124 | 2.30E-06 | 2.257 |
| MSMEG_0838 | 7.81E-04 | 2.242 |
| MSMEG_0559 | 3.31E-02 | 2.216 |
| MSMEG_3900 | 1.42E-02 | 2.139 |
| MSMEG_0572 | 4.31E-03 | 2.136 |
| MSMEG_3575 | 1.41E-04 | 2.111 |
| MSMEG_0633 | 2.06E-02 | 2.072 |
| MSMEG_6414 | 2.95E-02 | 2.022 |
| MSMEG_1634 | 4.18E-03 | 1.998 |
| MSMEG_5457 | 8.87E-03 | 1.989 |
| MSMEG_3628 | 8.12E-04 | 1.956 |
| MSMEG_0361 | 2.41E-04 | 1.919 |
| MSMEG_3212 | 2.80E-03 | 1.914 |
| MSMEG_1993 | 2.84E-02 | 1.749 |
| MSMEG_2982 | 2.80E-04 | 1.728 |
| MSMEG_2476 | 6.78E-04 | 1.681 |
| MSMEG_6187 | 6.39E-04 | 1.673 |
| MSMEG_6486 | 1.03E-05 | 1.668 |
| MSMEG_3265 | 3.98E-03 | 1.619 |
| MSMEG_4619 | 3.31E-02 | 1.570 |
| MSMEG_1991 | 1.04E-02 | 1.554 |
| MSMEG_3898 | 2.17E-02 | 1.553 |
| MSMEG_1060 | 1.71E-02 | 1.541 |
| MSMEG_5816 | 1.62E-02 | 1.535 |
| MSMEG_3170 | 9.21E-06 | 1.529 |
| MSMEG_6903 | 1.60E-02 | 1.499 |
| MSMEG_1465 | 2.13E-03 | 1.466 |
| MSMEG_3878 | 3.98E-03 | 1.465 |
| MSMEG_4624 | 1.68E-03 | 1.430 |
| MSMEG_2623 | 1.13E-02 | 1.429 |
| MSMEG_4884 | 3.98E-03 | 1.412 |
| MSMEG_5523 | 1.52E-02 | 1.378 |
| MSMEG_5489 | 1.16E-03 | 1.369 |
| MSMEG_0457 | 9.50E-03 | 1.365 |
| MSMEG_5652 | 9.50E-03 | 1.363 |
| MSMEG_5857 | 1.26E-02 | 1.345 |
| MSMEG_2661 | 4.41E-03 | 1.340 |
| MSMEG_6605 | 2.12E-02 | 1.328 |
| MSMEG_4934 | 1.15E-04 | 1.312 |
| MSMEG_4209 | 2.06E-02 | 1.304 |
| MSMEG_6459 | 3.28E-03 | 1.294 |
| MSMEG_1760 | 1.20E-03 | 1.292 |
| MSMEG_6184 | 1.59E-02 | 1.275 |
| MSMEG_0128 | 1.37E-02 | 1.272 |
| MSMEG_0918 | 1.27E-02 | 1.268 |
| MSMEG_2389 | 3.18E-03 | 1.267 |
| MSMEG_5213 | 9.39E-03 | 1.266 |
| MSMEG_6002 | 3.12E-05 | 1.266 |
| MSMEG_3742 | 9.81E-03 | 1.255 |
| MSMEG_3264 | 1.26E-03 | 1.233 |
| MSMEG_1739 | 6.23E-03 | 1.222 |
| MSMEG_2735 | 8.87E-03 | 1.221 |
| MSMEG_3534 | 1.55E-02 | 1.205 |
| MSMEG_6945 | 1.67E-02 | 1.200 |
| MSMEG_0814 | 3.19E-04 | 1.198 |
| MSMEG_1109 | 7.23E-03 | 1.187 |
| MSMEG_0243 | 1.91E-05 | 1.186 |
| MSMEG_5726 | 3.65E-02 | 1.183 |
| MSMEG_0782 | 4.71E-02 | 1.177 |
| MSMEG_2792 | 3.19E-03 | 1.175 |
| MSMEG_3899 | 1.75E-02 | 1.173 |
| MSMEG_3113 | 1.53E-02 | 1.170 |
| MSMEG_1875 | 5.90E-03 | 1.165 |
| MSMEG_0551 | 1.21E-02 | 1.157 |
| MSMEG_4516 | 6.13E-04 | 1.157 |
| MSMEG_5999 | 7.49E-03 | 1.154 |
| MSMEG_3271 | 3.28E-03 | 1.153 |
| MSMEG_6180 | 9.56E-03 | 1.148 |
| MSMEG_6399 | 9.48E-05 | 1.138 |
| MSMEG_0795 | 1.50E-03 | 1.125 |
| MSMEG_0892 | 1.57E-03 | 1.112 |
| MSMEG_5941 | 1.15E-02 | 1.111 |
| MSMEG_3193 | 3.19E-02 | 1.109 |
| MSMEG_1918 | 3.70E-02 | 1.093 |
| MSMEG_0406 | 9.36E-05 | 1.092 |
| MSMEG_6373 | 1.71E-03 | 1.091 |
| MSMEG_5032 | 9.81E-03 | 1.090 |
| MSMEG_5838 | 3.38E-03 | 1.086 |
| MSMEG_3119 | 6.63E-03 | 1.081 |
| MSMEG_3278 | 9.81E-03 | 1.060 |
| MSMEG_1705 | 2.30E-02 | 1.058 |
| MSMEG_2227 | 3.62E-03 | 1.058 |
| MSMEG_2078 | 2.73E-04 | 1.043 |
| MSMEG_5087 | 2.08E-02 | 1.042 |
| MSMEG_4297 | 8.87E-03 | 1.039 |
| MSMEG_5728 | 1.02E-03 | 1.035 |
| MSMEG_1115 | 1.79E-04 | 1.029 |
| MSMEG_3788 | 6.36E-03 | 1.025 |
| MSMEG_4231 | 1.35E-03 | 1.025 |
| MSMEG_5922 | 7.62E-03 | 1.017 |
| MSMEG_3422 | 2.68E-02 | 1.008 |
| MSMEG_2472 | 3.03E-02 | 1.002 |
| MSMEG_3360 | 4.33E-02 | 1.001 |
| MSMEG_5660 | 1.42E-02 | 1.000 |
| MSMEG_1414 | 1.63E-02 | 0.999 |
| MSMEG_6894 | 1.92E-03 | 0.993 |
| MSMEG_1474 | 1.77E-02 | 0.990 |
| MSMEG_3864 | 1.17E-02 | 0.986 |
| MSMEG_0829 | 5.17E-03 | 0.983 |
| MSMEG_1140 | 1.63E-02 | 0.982 |
| MSMEG_1073 | 1.17E-02 | 0.981 |
| MSMEG_5771 | 9.65E-03 | 0.980 |
| MSMEG_4181 | 5.84E-03 | 0.977 |
| MSMEG_6110 | 1.26E-02 | 0.976 |
| MSMEG_5055 | 9.61E-03 | 0.974 |
| MSMEG_5062 | 2.61E-03 | 0.969 |
| MSMEG_6186 | 6.79E-03 | 0.962 |
| MSMEG_2536 | 8.07E-03 | 0.953 |
| MSMEG_6875 | 1.98E-03 | 0.953 |
| MSMEG_3061 | 9.50E-03 | 0.951 |
| MSMEG_3142 | 2.28E-02 | 0.950 |
| MSMEG_2895 | 1.59E-02 | 0.948 |
| MSMEG_5201 | 1.79E-02 | 0.942 |
| MSMEG_3775 | 3.66E-02 | 0.942 |
| MSMEG_2512 | 4.98E-02 | 0.936 |
| MSMEG_2252 | 1.28E-03 | 0.926 |
| MSMEG_5931 | 6.13E-04 | 0.908 |
| MSMEG_5259 | 1.48E-02 | 0.903 |
| MSMEG_4937 | 8.68E-04 | 0.897 |
| MSMEG_0840 | 6.96E-04 | 0.895 |
| MSMEG_2614 | 4.14E-02 | 0.888 |
| MSMEG_3094 | 3.80E-03 | 0.880 |
| MSMEG_3045 | 1.04E-02 | 0.876 |
| MSMEG_2622 | 2.33E-02 | 0.873 |
| MSMEG_0304 | 2.44E-02 | 0.871 |
| MSMEG_3319 | 3.45E-02 | 0.869 |
| MSMEG_0894 | 3.95E-02 | 0.868 |
| MSMEG_4334 | 1.02E-02 | 0.868 |
| MSMEG_3580 | 9.41E-03 | 0.860 |
| MSMEG_1444 | 1.25E-03 | 0.853 |
| MSMEG_4935 | 5.56E-03 | 0.853 |
| MSMEG_6137 | 1.63E-02 | 0.850 |
| MSMEG_3026 | 1.52E-02 | 0.849 |
| MSMEG_6208 | 1.16E-03 | 0.848 |
| MSMEG_6926 | 1.71E-03 | 0.843 |
| MSMEG_3210 | 1.47E-03 | 0.841 |
| MSMEG_4198 | 3.32E-02 | 0.836 |
| MSMEG_2115 | 2.21E-02 | 0.830 |
| MSMEG_3879 | 2.26E-02 | 0.829 |
| MSMEG_5248 | 9.81E-03 | 0.827 |
| MSMEG_2123 | 5.17E-03 | 0.826 |
| MSMEG_0595 | 1.61E-02 | 0.825 |
| MSMEG_2224 | 3.98E-03 | 0.824 |
| MSMEG_6900 | 4.16E-03 | 0.820 |
| MSMEG_1521 | 2.75E-02 | 0.818 |
| MSMEG_0750 | 2.06E-02 | 0.817 |
| MSMEG_0760 | 1.81E-03 | 0.816 |
| MSMEG_4917 | 4.66E-03 | 0.814 |
| MSMEG_5732 | 1.33E-02 | 0.814 |
| MSMEG_4253 | 2.53E-02 | 0.810 |
| MSMEG_1157 | 3.14E-03 | 0.809 |
| MSMEG_4940 | 5.09E-03 | 0.809 |
| MSMEG_5664 | 5.71E-04 | 0.798 |
| MSMEG_1510 | 2.61E-02 | 0.796 |
| MSMEG_3619 | 1.81E-03 | 0.796 |
| MSMEG_3478 | 3.20E-03 | 0.792 |
| MSMEG_2359 | 8.87E-03 | 0.790 |
| MSMEG_3970 | 4.93E-03 | 0.787 |
| MSMEG_5454 | 1.04E-02 | 0.786 |
| MSMEG_0929 | 3.01E-02 | 0.780 |
| MSMEG_0221 | 2.78E-02 | 0.777 |
| MSMEG_6602 | 1.71E-03 | 0.772 |
| MSMEG_2440 | 2.09E-02 | 0.770 |
| MSMEG_4571 | 7.14E-03 | 0.769 |
| MSMEG_1704 | 3.17E-03 | 0.767 |
| MSMEG_5262 | 1.03E-02 | 0.763 |
| MSMEG_2112 | 1.98E-02 | 0.752 |
| MSMEG_0392 | 2.28E-02 | 0.751 |
| MSMEG_6398 | 1.44E-02 | 0.749 |
| MSMEG_6037 | 1.41E-02 | 0.748 |
| MSMEG_5490 | 4.85E-03 | 0.743 |
| MSMEG_3042 | 2.86E-03 | 0.740 |
| MSMEG_6289 | 2.22E-02 | 0.739 |
| MSMEG_2752 | 3.61E-03 | 0.735 |
| MSMEG_0539 | 1.13E-02 | 0.728 |
| MSMEG_6003 | 4.92E-02 | 0.727 |
| MSMEG_1120 | 4.85E-03 | 0.727 |
| MSMEG_3863 | 1.43E-02 | 0.725 |
| MSMEG_1947 | 3.45E-02 | 0.712 |
| MSMEG_1931 | 2.90E-02 | 0.710 |
| MSMEG_5318 | 3.40E-02 | 0.701 |
| MSMEG_5727 | 1.16E-02 | 0.700 |
| MSMEG_4271 | 1.13E-02 | 0.696 |
| MSMEG_4454 | 1.68E-03 | 0.696 |
| MSMEG_3036 | 4.49E-02 | 0.694 |
| MSMEG_2121 | 1.14E-02 | 0.690 |
| MSMEG_1882 | 3.65E-02 | 0.689 |
| MSMEG_2456 | 1.29E-02 | 0.689 |
| MSMEG_3073 | 3.28E-03 | 0.683 |
| MSMEG_2654 | 9.96E-03 | 0.683 |
| MSMEG_1376 | 2.71E-02 | 0.682 |
| MSMEG_2658 | 1.92E-03 | 0.681 |
| MSMEG_5495 | 1.99E-02 | 0.678 |
| MSMEG_1925 | 2.61E-02 | 0.675 |
| MSMEG_6311 | 2.49E-02 | 0.674 |
| MSMEG_5371 | 3.69E-02 | 0.673 |
| MSMEG_0209 | 2.68E-02 | 0.664 |
| MSMEG_2079 | 3.98E-03 | 0.661 |
| MSMEG_0638 | 1.59E-02 | 0.658 |
| MSMEG_4936 | 3.38E-03 | 0.658 |
| MSMEG_4505 | 3.22E-02 | 0.656 |
| MSMEG_5330 | 1.29E-02 | 0.655 |
| MSMEG_6285 | 1.15E-02 | 0.652 |
| MSMEG_3347 | 1.45E-02 | 0.647 |
| MSMEG_1039 | 8.90E-03 | 0.645 |
| MSMEG_4939 | 3.38E-03 | 0.642 |
| MSMEG_2060 | 4.88E-02 | 0.636 |
| MSMEG_3872 | 2.65E-02 | 0.635 |
| MSMEG_4997 | 9.50E-03 | 0.635 |
| MSMEG_4275 | 1.43E-02 | 0.635 |
| MSMEG_4974 | 6.78E-03 | 0.635 |
| MSMEG_0969 | 3.12E-02 | 0.633 |
| MSMEG_2695 | 1.79E-02 | 0.632 |
| MSMEG_1051 | 4.31E-03 | 0.632 |
| MSMEG_0697 | 2.35E-02 | 0.625 |
| MSMEG_5890 | 3.17E-03 | 0.625 |
| MSMEG_6374 | 3.12E-02 | 0.624 |
| MSMEG_0862 | 6.30E-03 | 0.624 |
| MSMEG_5995 | 6.30E-03 | 0.623 |
| MSMEG_6098 | 1.92E-02 | 0.622 |
| MSMEG_1443 | 3.55E-02 | 0.620 |
| MSMEG_6754 | 1.61E-02 | 0.619 |
| MSMEG_4595 | 3.12E-02 | 0.618 |
| MSMEG_5932 | 2.16E-02 | 0.617 |
| MSMEG_5492 | 1.81E-03 | 0.615 |
| MSMEG_3418 | 3.05E-03 | 0.615 |
| MSMEG_1436 | 7.45E-03 | 0.611 |
| MSMEG_5724 | 1.05E-02 | 0.608 |
| MSMEG_2765 | 1.63E-02 | 0.607 |
| MSMEG_5066 | 2.84E-02 | 0.607 |
| MSMEG_2081 | 2.17E-02 | 0.606 |
| MSMEG_4270 | 4.31E-03 | 0.603 |
| MSMEG_0114 | 1.55E-02 | 0.603 |
| MSMEG_2376 | 2.28E-02 | 0.600 |
| MSMEG_6054 | 4.11E-02 | 0.598 |
| MSMEG_4494 | 1.43E-02 | 0.597 |
| MSMEG_0854 | 2.06E-02 | 0.596 |
| MSMEG_1440 | 3.18E-02 | 0.590 |
| MSMEG_1527 | 4.17E-02 | 0.589 |
| MSMEG_0786 | 1.96E-02 | 0.589 |
| MSMEG_2964 | 9.94E-03 | 0.586 |
| MSMEG_5721 | 1.18E-03 | 0.586 |

**Supplementary Table 5**

List of proteins depleted during growth in the absence of glycerol in *M. smegmatis* Δ*cpa* as compared to its parent strain by label-free quantification mass spectrometry

| **Locus tag** | **p-value (q.mod)** | **log_2_(∆/WT)** |
| --- | --- | --- |
| MSMEG_0334 | 2.90E-07 | -5.555 |
| MSMEG_0314 | 1.02E-07 | -5.383 |
| MSMEG_1193 | 4.05E-08 | -4.867 |
| MSMEG_6201 | 9.03E-08 | -4.774 |
| MSMEG_6645 | 2.21E-03 | -4.172 |
| MSMEG_2935 | 1.57E-06 | -3.808 |
| MSMEG_5970 | 2.90E-02 | -3.789 |
| MSMEG_0826 | 8.61E-04 | -3.656 |
| MSMEG_5209 | 4.47E-06 | -3.469 |
| MSMEG_2719 | 3.27E-03 | -3.390 |
| MSMEG_6700 | 7.33E-06 | -3.060 |
| MSMEG_1005 | 2.28E-02 | -3.053 |
| MSMEG_2723 | 3.18E-03 | -3.041 |
| MSMEG_6073 | 7.27E-06 | -2.919 |
| MSMEG_4396 | 4.10E-02 | -2.874 |
| MSMEG_3132 | 3.08E-03 | -2.855 |
| MSMEG_3708 | 2.06E-02 | -2.845 |
| MSMEG_1978 | 3.62E-02 | -2.693 |
| MSMEG_4042 | 4.31E-03 | -2.628 |
| MSMEG_5220 | 9.84E-06 | -2.595 |
| MSMEG_1812 | 5.54E-03 | -2.496 |
| MSMEG_5002 | 3.59E-04 | -2.464 |
| MSMEG_3430 | 3.41E-06 | -2.459 |
| MSMEG_5582 | 1.20E-04 | -2.444 |
| MSMEG_0194 | 8.09E-03 | -2.418 |
| MSMEG_6302 | 1.57E-03 | -2.386 |
| MSMEG_4589 | 4.93E-02 | -2.325 |
| MSMEG_0158 | 3.31E-04 | -2.324 |
| MSMEG_0240 | 3.98E-03 | -2.204 |
| MSMEG_5274 | 3.87E-05 | -2.179 |
| MSMEG_6627 | 4.20E-02 | -2.136 |
| MSMEG_0746 | 1.42E-02 | -2.093 |
| MSMEG_4238 | 2.18E-04 | -1.990 |
| MSMEG_2198 | 1.45E-03 | -1.973 |
| MSMEG_0762 | 1.89E-03 | -1.962 |
| MSMEG_1952 | 3.98E-03 | -1.937 |
| MSMEG_6291 | 8.00E-04 | -1.932 |
| MSMEG_6422 | 2.99E-04 | -1.902 |
| MSMEG_1254 | 5.54E-03 | -1.897 |
| MSMEG_0933 | 5.90E-03 | -1.896 |
| MSMEG_5632 | 2.93E-04 | -1.887 |
| MSMEG_6936 | 6.64E-05 | -1.878 |
| MSMEG_2271 | 5.96E-05 | -1.868 |
| MSMEG_1680 | 7.27E-06 | -1.839 |
| MSMEG_0926 | 1.05E-02 | -1.833 |
| MSMEG_1679 | 1.40E-02 | -1.820 |
| MSMEG_4976 | 3.64E-04 | -1.798 |
| MSMEG_1494 | 1.03E-05 | -1.751 |
| MSMEG_3477 | 5.24E-03 | -1.741 |
| MSMEG_5221 | 7.72E-03 | -1.713 |
| MSMEG_2939 | 1.17E-03 | -1.705 |
| MSMEG_0057 | 4.53E-02 | -1.691 |
| MSMEG_4076 | 2.06E-02 | -1.685 |
| MSMEG_0916 | 8.06E-04 | -1.679 |
| MSMEG_0938 | 4.60E-02 | -1.675 |
| MSMEG_2919 | 1.60E-04 | -1.673 |
| MSMEG_6292 | 2.91E-03 | -1.671 |
| MSMEG_3615 | 1.61E-02 | -1.667 |
| MSMEG_6859 | 2.78E-02 | -1.658 |
| MSMEG_6249 | 4.05E-02 | -1.616 |
| MSMEG_4567 | 8.67E-04 | -1.607 |
| MSMEG_3488 | 3.20E-03 | -1.607 |
| MSMEG_0608 | 1.20E-04 | -1.586 |
| MSMEG_6649 | 1.10E-02 | -1.558 |
| MSMEG_4743 | 1.72E-02 | -1.547 |
| MSMEG_3489 | 1.69E-04 | -1.532 |
| MSMEG_2659 | 2.49E-02 | -1.520 |
| MSMEG_0592 | 3.98E-03 | -1.519 |
| MSMEG_4985 | 9.91E-03 | -1.513 |
| MSMEG_1682 | 3.86E-04 | -1.511 |
| MSMEG_0744 | 4.33E-03 | -1.510 |
| MSMEG_1033 | 1.35E-03 | -1.481 |
| MSMEG_5935 | 1.73E-03 | -1.466 |
| MSMEG_5792 | 1.18E-05 | -1.461 |
| MSMEG_5300 | 4.47E-06 | -1.457 |
| MSMEG_0318 | 7.49E-03 | -1.451 |
| MSMEG_5022 | 1.57E-03 | -1.442 |
| MSMEG_0441 | 1.23E-04 | -1.432 |
| MSMEG_3041 | 3.45E-02 | -1.432 |
| MSMEG_1575 | 1.31E-03 | -1.414 |
| MSMEG_1946 | 1.45E-02 | -1.380 |
| MSMEG_3644 | 6.57E-05 | -1.375 |
| MSMEG_3449 | 2.78E-02 | -1.363 |
| MSMEG_0409 | 2.71E-02 | -1.362 |
| MSMEG_2299 | 6.58E-05 | -1.349 |
| MSMEG_4921 | 3.71E-03 | -1.336 |
| MSMEG_2580 | 2.38E-03 | -1.332 |
| MSMEG_3743 | 1.45E-03 | -1.327 |
| MSMEI_0386 | 9.36E-05 | -1.323 |
| MSMEG_0224 | 7.94E-05 | -1.320 |
| MSMEG_1317 | 2.17E-02 | -1.312 |
| MSMEG_6106 | 9.36E-05 | -1.309 |
| MSMEG_4394 | 1.25E-03 | -1.265 |
| MSMEG_4687 | 4.60E-02 | -1.258 |
| MSMEG_2779 | 4.97E-02 | -1.244 |
| MSMEG_0824 | 3.74E-04 | -1.237 |
| MSMEG_1927 | 1.36E-04 | -1.221 |
| MSMEG_1970 | 1.68E-03 | -1.212 |
| MSMEG_4686 | 3.12E-02 | -1.209 |
| MSMEG_0411 | 6.41E-04 | -1.204 |
| MSMEG_3883 | 2.17E-04 | -1.192 |
| MSMEG_2405 | 1.47E-03 | -1.183 |
| MSMEG_6338 | 9.22E-03 | -1.162 |
| MSMEG_5680 | 1.16E-03 | -1.161 |
| MSMEG_4222 | 3.87E-03 | -1.156 |
| MSMEG_4665 | 3.87E-03 | -1.156 |
| MSMEG_4557 | 5.34E-03 | -1.148 |
| MSMEG_2470 | 2.68E-02 | -1.143 |
| MSMEG_6935 | 2.51E-02 | -1.141 |
| MSMEG_5789 | 2.07E-03 | -1.139 |
| MSMEG_3988 | 3.22E-02 | -1.132 |
| MSMEG_2084 | 4.67E-02 | -1.128 |
| MSMEG_3141 | 1.82E-02 | -1.111 |
| MSMEG_1594 | 2.06E-02 | -1.110 |
| MSMEG_1314 | 9.75E-03 | -1.109 |
| MSMEG_5593 | 7.19E-03 | -1.099 |
| MSMEG_6939 | 4.70E-02 | -1.093 |
| MSMEG_4668 | 5.16E-03 | -1.088 |
| MSMEG_3201 | 2.48E-02 | -1.086 |
| MSMEG_0434 | 8.06E-05 | -1.083 |
| MSMEG_0203 | 1.34E-02 | -1.082 |
| MSMEG_2395 | 2.58E-02 | -1.073 |
| MSMEG_4284 | 7.49E-03 | -1.072 |
| MSMEG_1516 | 3.96E-04 | -1.070 |
| MSMEG_0825 | 1.35E-03 | -1.068 |
| MSMEG_6541 | 5.69E-04 | -1.063 |
| MSMEG_2667 | 4.18E-04 | -1.038 |
| MSMEG_6239 | 2.17E-02 | -1.036 |
| MSMEG_6662 | 3.84E-03 | -1.030 |
| MSMEG_0882 | 2.06E-02 | -1.018 |
| MSMEG_3667 | 1.63E-02 | -1.011 |
| MSMEG_4899 | 9.81E-03 | -1.009 |
| MSMEG_3768 | 4.33E-03 | -1.007 |
| MSMEG_0530 | 1.57E-03 | -1.006 |
| MSMEG_2128 | 1.97E-02 | -1.000 |
| MSMEG_3472 | 1.14E-03 | -0.992 |
| MSMEG_4125 | 1.10E-03 | -0.983 |
| MSMEG_1369 | 2.06E-02 | -0.975 |
| MSMEG_2606 | 3.95E-02 | -0.970 |
| MSMEG_4669 | 6.02E-03 | -0.965 |
| MSMEG_0252 | 3.98E-03 | -0.962 |
| MSMEG_4342 | 2.13E-02 | -0.961 |
| MSMEG_3829 | 4.48E-02 | -0.951 |
| MSMEG_5554 | 2.41E-04 | -0.949 |
| MSMEG_5073 | 7.81E-04 | -0.936 |
| MSMEG_2450 | 3.38E-03 | -0.933 |
| MSMEG_2035 | 1.26E-03 | -0.921 |
| MSMEG_5681 | 3.07E-02 | -0.916 |
| MSMEG_1205 | 2.41E-04 | -0.916 |
| MSMEG_0670 | 3.65E-02 | -0.908 |
| MSMEG_4560 | 2.17E-02 | -0.902 |
| MSMEG_4827 | 3.65E-02 | -0.900 |
| MSMEG_3605 | 1.79E-02 | -0.900 |
| MSMEG_0435 | 5.40E-03 | -0.894 |
| MSMEG_3335 | 1.31E-03 | -0.891 |
| MSMEG_5800 | 4.15E-03 | -0.889 |
| MSMEG_2768 | 2.61E-02 | -0.888 |
| MSMEG_1047 | 1.14E-03 | -0.884 |
| MSMEG_3460 | 3.81E-02 | -0.882 |
| MSMEG_3396 | 1.26E-02 | -0.881 |
| MSMEG_4330 | 5.73E-03 | -0.877 |
| MSMEG_0791 | 2.43E-03 | -0.851 |
| MSMEG_5050 | 4.31E-03 | -0.851 |
| MSMEG_0388 | 1.97E-02 | -0.850 |
| MSMEG_0688 | 6.63E-03 | -0.840 |
| MSMEG_0157 | 4.48E-02 | -0.835 |
| MSMEG_6759 | 4.18E-04 | -0.832 |
| MSMEG_3846 | 3.38E-03 | -0.827 |
| MSMEG_1825 | 1.71E-02 | -0.825 |
| MSMEG_2923 | 5.17E-03 | -0.824 |
| MSMEG_0400 | 1.08E-03 | -0.824 |
| MSMEG_3302 | 1.53E-02 | -0.823 |
| MSMEG_5858 | 2.51E-02 | -0.820 |
| MSMEG_3317 | 1.75E-02 | -0.818 |
| MSMEG_0836 | 6.11E-03 | -0.817 |
| MSMEG_1426 | 5.24E-03 | -0.815 |
| MSMEG_2201 | 2.06E-02 | -0.807 |
| MSMEG_4903 | 2.17E-02 | -0.807 |
| MSMEG_3059 | 9.81E-03 | -0.804 |
| MSMEG_6575 | 1.48E-02 | -0.792 |
| MSMEG_4565 | 1.71E-03 | -0.792 |
| MSMEG_2743 | 2.01E-02 | -0.787 |
| MSMEG_6431 | 1.31E-03 | -0.786 |
| MSMEG_6085 | 7.86E-03 | -0.785 |
| MSMEG_0100 | 1.71E-02 | -0.784 |
| MSMEG_1913 | 2.22E-02 | -0.783 |
| MSMEG_0003 | 3.44E-02 | -0.782 |
| MSMEG_1874 | 1.32E-03 | -0.781 |
| MSMEG_3211 | 5.88E-03 | -0.780 |
| MSMEG_3065 | 3.87E-03 | -0.777 |
| MSMEG_1029 | 7.86E-03 | -0.776 |
| MSMEG_2785 | 1.17E-02 | -0.773 |
| MSMEG_2061 | 2.37E-02 | -0.749 |
| MSMEG_5553 | 3.35E-02 | -0.749 |
| MSMEG_3822 | 9.61E-03 | -0.749 |
| MSMEG_5878 | 3.22E-02 | -0.748 |
| MSMEG_5475 | 2.73E-02 | -0.740 |
| MSMEG_6616 | 4.20E-02 | -0.739 |
| MSMEG_4313 | 4.88E-02 | -0.737 |
| MSMEG_1773 | 9.61E-03 | -0.734 |
| MSMEG_1204 | 2.31E-02 | -0.731 |
| MSMEG_2540 | 2.21E-03 | -0.725 |
| MSMEG_2399 | 2.49E-02 | -0.724 |
| MSMEG_1675 | 3.87E-03 | -0.722 |
| MSMEG_5222 | 1.81E-03 | -0.721 |
| MSMEG_3559 | 1.42E-02 | -0.713 |
| MSMEG_6733 | 3.61E-02 | -0.709 |
| MSMEG_3677 | 8.20E-04 | -0.708 |
| MSMEG_3034 | 2.25E-02 | -0.702 |
| MSMEG_0097 | 4.66E-02 | -0.701 |
| MSMEG_1984 | 3.55E-02 | -0.698 |
| MSMEG_6730 | 2.05E-02 | -0.696 |
| MSMEG_6082 | 1.81E-03 | -0.694 |
| MSMEG_3860 | 3.32E-02 | -0.692 |
| MSMEG_3799 | 9.81E-03 | -0.689 |
| MSMEG_3053 | 6.02E-03 | -0.683 |
| MSMEG_3535 | 1.71E-02 | -0.676 |
| MSMEG_0856 | 6.74E-03 | -0.675 |
| MSMEG_2778 | 6.21E-03 | -0.667 |
| MSMEG_4116 | 3.25E-02 | -0.660 |
| MSMEG_3471 | 2.26E-03 | -0.660 |
| MSMEG_0309 | 3.71E-03 | -0.660 |
| MSMEG_4261 | 2.13E-02 | -0.658 |
| MSMEG_5629 | 2.75E-02 | -0.655 |
| MSMEG_1388 | 1.17E-02 | -0.651 |
| MSMEG_1291 | 2.22E-02 | -0.645 |
| MSMEG_0029 | 6.40E-03 | -0.640 |
| MSMEG_6107 | 1.70E-02 | -0.637 |
| MSMEG_1681 | 1.30E-02 | -0.635 |
| MSMEG_5252 | 1.13E-02 | -0.634 |
| MSMEG_4914 | 4.13E-02 | -0.629 |
| MSMEG_5837 | 8.68E-04 | -0.629 |
| MSMEG_6475 | 9.56E-03 | -0.627 |
| MSMEG_2529 | 2.76E-03 | -0.626 |
| MSMEG_6804 | 2.06E-02 | -0.623 |
| MSMEG_4497 | 3.40E-02 | -0.622 |
| MSMEG_4115 | 3.17E-02 | -0.620 |
| MSMEG_5574 | 9.56E-03 | -0.618 |
| MSMEG_1368 | 6.79E-03 | -0.613 |
| MSMEG_3798 | 1.32E-02 | -0.606 |
| MSMEG_0401 | 1.05E-02 | -0.603 |
| MSMEG_5614 | 1.40E-02 | -0.601 |
| MSMEG_2387 | 1.84E-03 | -0.599 |
| MSMEG_0643 | 3.32E-02 | -0.596 |
| MSMEG_5797 | 1.47E-02 | -0.594 |
| MSMEG_5412 | 3.75E-03 | -0.593 |
| MSMEG_3228 | 4.54E-02 | -0.593 |
| MSMEG_3206 | 2.26E-02 | -0.590 |
| MSMEG_6337 | 4.33E-03 | -0.588 |
| MSMEG_0422 | 2.55E-03 | -0.588 |
| MSMEG_4833 | 9.81E-03 | -0.587 |
